# An Atypical Case of Congenital Erythropoietic Porphyria

**DOI:** 10.3390/genes12111828

**Published:** 2021-11-19

**Authors:** Bénédicte Sudrié-Arnaud, Marine Legendre, Sarah Snanoudj, Fanny Pelluard, Soumeya Bekri, Abdellah Tebani

**Affiliations:** 1 Department of Metabolic Biochemistry, Normandie University, UNIROUEN, INSERM U1245, CHU Rouen, 76000 Rouen, France; b.sudrie-Arnaud@chu-rouen.fr (B.S.-A.); sarah.snanoudj@chu-rouen.fr (S.S.); soumeya.bekri@chu-rouen.fr (S.B.); 2 Service de Génétique Médicale, CHU de Bordeaux, 33400 Bordeaux, France; marine.legendre@chu-bordeaux.fr; 3 Service d’Anatomopathologie, CHU de Bordeaux, 33400 Bordeaux, France; fanny.pelluard@chu-bordeaux.fr

**Keywords:** UROS, congenital erythropoietic porphyria, Next-Generation Sequencing, hydrops fetalis, bone abnormalities

## Abstract

Congenital erythropoietic porphyria (CEP, OMIM #606938) is a severe autosomal recessive inborn error of heme biosynthesis. This rare panethnic disease is due to a deficiency of uroporphyrinogen III synthase (or cosynthase). Subsequently, its substrate, the hydroxymethylbilane is subsequently converted into uroporphyrinogen I in a non-enzymatic manner. Of note, uroporphyrinogen I cannot be metabolized into heme and its accumulation in red blood cells results in intramedullary and intravascular hemolysis. The related clinical symptoms occur most frequently during antenatal or neonatal periods but may also appear in late adulthood. The main antenatal clinical presentation is a non-immune hydrops fetalis. We report here two cases of antenatal CEP deficiency and a review of the reported cases in the literature.

## 1. Introduction

**Figure 1:**
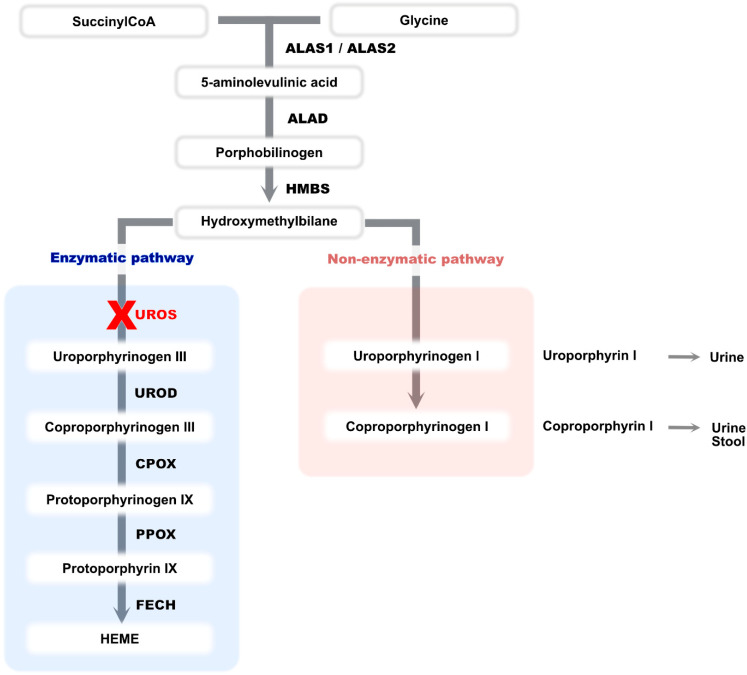
The human biosynthetic pathway of heme. ALAS: Aminolevulinic acid synthase, ALAD: Aminolevulinic acid dehydratase, HMBS: Hydroxymethylbilane synthase, UROS: Uroporphyrinogen III synthase, UROD: Uroporphyrinogen decarboxylase, CPOX: Coproporphyrinogen oxidase, PPOX: Protoporphyrinogen oxidase, FECH: Ferrochelatase.

Congenital erythropoietic porphyria (CEP, OMIM #606938) is a severe inborn error of heme biosynthesis autosomal recessive or X-linked trait due to mutations in *UROS* or *GATA1* genes [1]. Its prevalence is 1/1,000,000. It occurs most frequently during the antenatal period or just after birth but may also appear in late adulthood. This rare and panethnic disease is due to a severe deficiency of uroporphyrinogen III synthase (or cosynthase-UROS) [2]. This deficiency leads to an accumulation of hydroxymethylbilane which is subsequently converted into uroporphyrinogen I in a non-enzymatic manner. The latter cannot be metabolized into heme (Figure 1) [3]. Uroporphyrinogen I is therefore metabolized to coproporphyrinogen I, and both products accumulate in bone marrow, tissue, urine and feces [3,4]. The porphyrin accumulation in red blood cells results in intramedullary and intravascular hemolysis [5].

The main clinical features of CEP are severe photosensitivity which can lead to progressive photo-mutilation, blistering and friability of the skin in areas exposed to light, as well as a dark red coloration of urine during seizures, and hemolytic anemia. However, other signs can be found in moderate to severe forms such as corneal ulcerations, erythrodontia, or a defect in bone mineralization [3]. Current treatment involves sun avoidance. Blood transfusion is necessary for significant hemolysis. Bone marrow transplantation is the only cure for CEP and is considered in children with severe hematologic and cutaneous presentation.

Only eight cases with an antenatal presentation have been described in the literature [6,7,8,9,10]. This antenatal presentation is associated with hydrops fetalis and a polymalformative phenotype including a femur length lower than the third percentile, hyperechogenic bowels, pulmonary, cardiac and renal damage, facial dysmorphism, cytolysis and cholestasis. An elevation of uro- and coproporphyrin I isomers in the amniotic fluid which is generally dark reddish-brown has been observed [6].

We report here antenatal CEP deficiency in two siblings with an important polymalformative syndrome, which led to a diagnostic wandering. This case is of educational interest, and prompt to consider CEP diagnosis even in the absence of dark amniotic fluid.

## 2. Patients and Methods

### 2.1. Case Description

**Figure 2:**
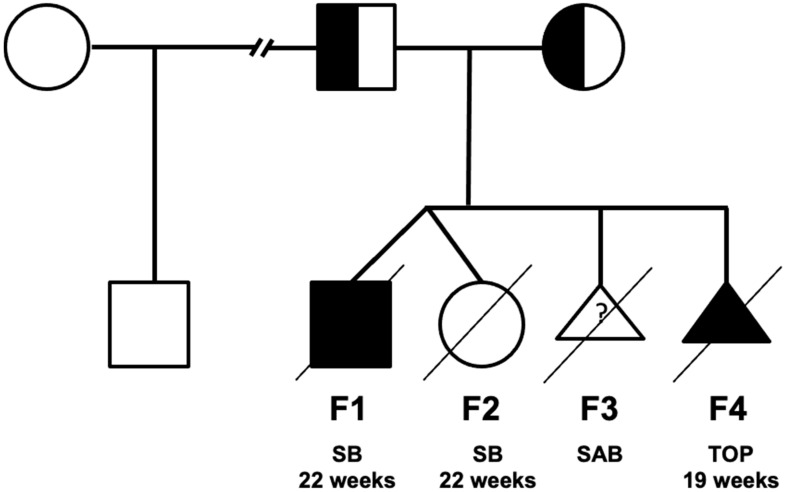
Family tree of the reported cases. SB: Stillbirth, SAB: spontaneous abortion, TOP: termination of pregnancy.

**Figure 3:**
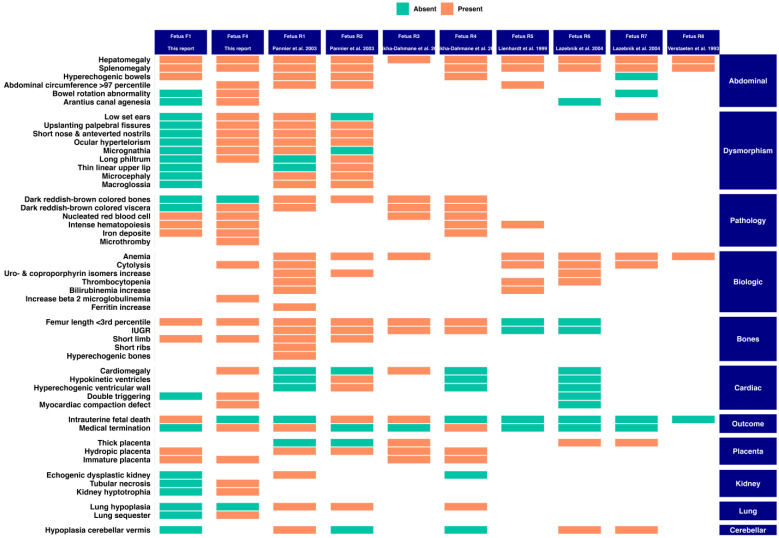
Overview of the clinical features of the two cases and the literature-reported cases [6,7,9,10].

A woman had a twin biamniotic pregnancy at the age of 39 (fetus F1 and F2 in Figure 2 and Figure 3, Supplementary Table S1). Upon the second trimester ultrasound, a male fetus (F1) was diagnosed with a hyperechogenic small intestine associated with short femurs resulting in a miscarriage at 22 weeks of amenorrhea for the twin pregnancy (the second fetus F2 was healthy). The F1 anatomopathological examination found short long bones, hydrops fetalis and splenomegaly. At the microscopic level, extra-medullary hematopoiesis and nucleated red blood cells with regenerative anemia associated with siderophilic pigments have been observed. One year later, a spontaneous miscarriage (fetus F3) was documented. One more year later, the first ultrasound in fetus F4 showed hygroma coli, ascitis, short femurs as well as a double outlet right ventricle. A family tree is presented in Figure 3. A medical termination of pregnancy was decided at 19 weeks of amenorrhea. The anatomopathological examination confirmed hydrops fetalis, hepatosplenomegaly, short long bones, abnormal bowel rotation, agenesis of the arantius canal, renal hypotrophy, pulmonary sequestration, dysmorphic features (short forehead, large nose, hypertelorism, long and stick philtrum, microretrognathism and low-set ears) and cardiac involvement with the right ventricle with double outlet. At the microscopic level, there were an intra and extrahepatic iron overload, microthrombi, extra-medullary hematopoiesis, proximal tubular necrosis, myocardial compaction defect, and moderate placental reticulocytosis. None of the fetuses presented dark brown tissue deposits at the anatomopathological examination. The etiological assessment showed normal hemoglobin electrophoresis in both parents. The F4 fetal biochemistry showed an increase in beta2 microglobulin (6.28 mg/L, (<5)), γ glutamyltransferase (GGT = 611 U/L, (14.8–34)) and aspartate aminotransferase (ASAT (Asprate Amino-Transferase) = 105 UI/L, (19.1–23.1)) and a decrease in albumin (17.2 g/L, (21–21.8)). This cholestasis and cytolysis were suggestive of a lysosomal disease (LD). However, the fetal biochemical assessment did not confirm it. Other pathologies have been also ruled out such as CDG-PMM2 (*PMM2*-congenital disorder of glycosylation), Smith–Lemli–Opitz and Blackfan–Diamond disease. At this point, a hydrops fetalis panel including inherited metabolic diseases (IMD) genes was requested [11].

### 2.2. Next Generation Sequencing

Fetal DNA samples issued from F1 and F4 were sequenced using the HydFet panel which includes 42 IMD-related genes known to be associated with hydrops fetalis [9]. This panel was implemented on an Illumina platform (San Diego, CA, USA), and was designed using the Agilent SureDesign Software 7.6 (Agilent Technologies Inc, Santa Clara, CA, USA). Coding regions and +/− 50 bp within flanking intronic sequences were targeted (121, 615 bases, 460 regions). Library construction was carried out using SureSelect QXT (Agilent Technologies Inc., Santa Clara, CA, USA) and sequencing was performed on NextSeq instruments (Illumina, San Diego, CA, USA) using 2 × 150 bp paired-end sequencing. Analysis was performed using a bioinformatics pipeline that included CASAVA suite v1.8 (Illumina, San Diego, CA, USA) and BWA-GATK 2.2.5 (Genome analysis ToolKit, Broad Institute, Cambridge, MA, USA) in parallel for mapping and variant calling, alamut batch (Interactive Biosoftware^®^, ROUEN, France) for variant annotation, and the in-house database CanDiD for prioritizing and filtering variant of interest. For the detection of copy number variants such as deletions or duplication, the CANOES algorithm was used [12,13].

## 3. Results

The HydFet panel results revealed a missense variant located in exon 4 of *UROS* gene, NM_000375.2: c.217T > C, (p.Cys73Arg) at a homozygous status. Depth mean was at 456 and coverage was at 100%. This variant has been considered pathogenic (class V) according to the American College of Medical Genetics (ACMG) recommendations [14]. This variant has been described in the literature and in ClinVar as pathogenic [15]. Its frequency in the general population is low (MAF ≤ 0.001, 1000 Genome Project phase 3) and the physico-chemical distance between cysteine and arginine is high. This variant accounts for 30% of alleles causing pathology [3]. The heterozygous carrier status of both parents was confirmed. 

## 4. Discussion

The reported cases present with an antenatal presentation of CEP including multisystemic alterations (Figure 2, Fetuses F1 and F4). We compared the clinical features characterized in these two sibling fetuses to those of the previously reported cases (Figure 3). Unlike the two cases of Pannier et al. whose echographic and anatomopathological signs are closest to our cases, the amniotic fluid in both pregnancies was not dark and the dark deposits are only found in the fetus (F4) at the pancreatic parenchyma level, which are two criteria that allow the diagnosis to be made before genetic analysis. The polymalformative phenotype of these fetuses is rarely described in the literature. On the other hand, the eight antenatal phenotypes already described have similar clinical signs, in particular: a femur length lower than the third percentile (R1 to R4), hyperechogenic bowels (R1, R2 and R4), pulmonary, cardiac and renal damage (R1 to R4) [6,7,8]. The facial dysmorphism found in fetus F4 has only been described in three fetuses (Fetuses R1, R2, and R7) but the morphological anomalies (hypertelorism, long philtrum, micrognathism and low-set ears) are similar [6]. Similarly, cytolysis and cholestasis have also been described in Fetuses R1, R5, R6 and R7 (Supplementary Table S1) [6,8]. Of note, hepatomegaly has been observed in all cases. Furthermore, splenomegaly is sometimes found in older patients in response to the increased uptake of abnormal erythrocytes coming from the blood stream. The hydrops fetalis might be explained by in utero hemolytic anemia [3].

Interestingly, these different cases highlight common aspects between LD and CEP [5]. Indeed, there are many similar clinical features such as short limbs, hepatosplenomegaly, cardiac damage, dysmorphia or even hydrops fetalis. These LD-related damages are mostly due to an accumulation of non-degraded macromolecules in the lysosome [16]. The suggestive hypothesis is that polymalformative syndrome, in these cases, is secondary to an accumulation of porphyrins (uro- and coproporphyrins) in the tissues, leading to these multi-visceral damages. The affected bones have shown abundant brown coloration and are fluorescent under an ultraviolet lamp in all these reported cases [6,7,8]. Furthermore, bone involvement has been also reported in adult patients. Indeed, the accumulation of porphyrins in the bone causes its demineralization and the shortening of the bones can also be explained by the contraction of the joints secondary to the scleroderma [3]. Furthermore, porphyrins toxicity through reactive oxygen species (ROS) generation leading to significant oxidative stress and DNA damage might be an exploration avenue.

It should be noted that apart from the R5, R6 and R7 fetus, the reported literature of *UROS* sequencing showed only the presence of c.217T > C, (p.Cys73Arg) variant at a heterozygous state, the other variant was not found [6,7]. Given the total loss of enzymatic activity and the phenotype severity, the authors believe that the second variant could be found in non-sequenced regions (promoter, or intronic sequences) [6]. This identified variant (c.217T > C, (p.Cys73Arg)) led to protein misfolding resulting in an enzymatic activity lower than 1% [3].

## 5. Conclusions

The reported cases highlight the clinical relevance and the need for a dedicated hydrops fetalis panel (HydFet) in the etiological investigation of undiagnosed non-immune hydrops fetalis. Indeed, the ultrasound and anatomopathological features such as dark brown tissue deposits are not always sufficient in congenital erythropoietic porphyria with antenatal onset and may lead to a diagnostic wavering. It is therefore important to regularly update the panel gene list and add all the new documented etiologies that can lead to a hydrops fetalis. This also highlights the paradigm shift in IMD investigations using integrative methods.

## Data Availability

All the data that support the findings are presented in the manuscript and the Supplementary Materials.

## Supplementary Materials

The following are available online at https://www.mdpi.com/article/10.3390/genes12111828/s1, Table S1: Clinical data of UROS deficiency with fetal presentation.
